# Correlation of molecular human leukocyte antigen typing and outcome in high-risk melanoma patients receiving adjuvant interferon

**DOI:** 10.1002/cncr.25211

**Published:** 2010-06-14

**Authors:** Helen Gogas, John M Kirkwood, Christine S Falk, Urania Dafni, Vernon K Sondak, Dimosthenis Tsoutsos, Alexandros Stratigos, Christos Markopoulos, Dimitrios Pectasides, Maria Spyropoulou-Vlachou

**Affiliations:** 1 First Department of Medicine, University of Athens, Medical SchoolAthens, Greece; 2 University of Pittsburgh Cancer Institute, Hillman Cancer CenterPittsburgh, Pennsylvania; 3 German Cancer Research Center, National Center for Tumor Diseases, National Center for Tumor Diseases, Institute for Immunology, University of HeidelbergHeidelberg, Germany; 4 Laboratory of Biostatistics, University of Athens School of NursingAthens, Greece; 5 H. Lee Moffitt Cancer Center and Research Institute, University of South FloridaTampa, Florida; 6 Department of Plastic Surgery and Microsurgery, G. Gennimatas General Hospital of AthensAthens, Greece; 7 Department of Dermatology, University of Athens, Andreas Sygros HospitalAthens, Greece; 8 Second Department of Internal Medicine-Propaedeutic, Oncology Section, University General Hospital AttikonAthens, Greece; 9 Department of Immunology, National Tissue Typing Center, General Hospital of AthensAthens, Greece

**Keywords:** human leukocyte antigen, melanoma, adjuvant interferon, prediction

## Abstract

**BACKGROUND:**

Interferon is approved for adjuvant treatment of patients with stage IIB/III melanoma. The identification of predictive markers that would permit selection of patients would be beneficial. Specific human leukocyte antigen (HLA) class I and II antigens have previously shown an association with response to therapy or overall survival of patients with metastatic melanoma.

**METHODS:**

A total of 284 high-risk melanoma patients participating in a randomized trial and 246 healthy controls were molecularly typed for HLA class I and II. Specific allele frequencies were compared between the healthy and patient populations, as well as presence or absence of these in relation to recurrence. Alleles related to autoimmune disease were also investigated.

**RESULTS:**

No significant differences were found between the distribution of HLA genotype in the melanoma population compared with healthy controls. Correlations between nonrecurrence and the presence of HLA-Cw*06 allele were noted present in 19.3% of melanoma patients. The median relapse-free survival of the Cw*06-positive cohort was 100.2 months versus 37.3 months in the Cw*06-negative cohort (*P* = .013). The median overall survival for the Cw*06-positive cohort has not yet been reached, versus 78.9 months in the Cw*06-negative cohort (*P* = .025). HLA-Cw*06 was present in 29.79% of patients in the autoimmunity group and 15.38% of patients in the nonautoimmunity group (*P* = .049).

**CONCLUSIONS:**

No allele was associated with absence of recurrence in patients receiving adjuvant interferon with the exception of HLA-Cw*06, an allele correlated with psoriasis. HLA-Cw*06-positive patients have better relapse-free and overall survival. Cancer 2010. © 2010 American Cancer Society.

Patients with stage IIB, IIC, and III melanoma as defined by the American Joint Committee on Cancer (AJCC sixth edition) are characterized as a group at high risk, with relapse and mortality risks exceeding 40% at 5 years.^1^ Several large cooperative group trials have evaluated adjuvant therapy with high-dose interferon (IFN)-α2b in this patient population and have consistently demonstrated significant prolongation of relapse-free survival (RFS) as compared with observation or GM2 coupled to keyhole limpet hemocyanin (KLH) and combined with the QS-21 adjuvant (GMK)-vaccine.^2-4^ Two of these trials have also demonstrated a significant improvement in overall survival (OS).^2^,^4^ The pooled analysis of 4 Eastern Cooperative Oncology Group and intergroup trials conducted between 1984 and 2001 has shown a durable impact of high-dose IFN on RFS, but not on OS.^5^ Analyses of the RFS and OS curves from the initial trial (E1684) revealed early separation between the high-dose IFN-α2b and the observation arms.^2^ Therefore, the 1-month intravenous induction phase of the regimen may be necessary and sufficient to reduce the risk of recurrence, and several prospective trials designed to test this hypothesis are currently ongoing or have recently been reported.^6^

Unfortunately, acceptance of high-dose IFN-α2b has been limited because of the toxicity and cost of this regimen and the lack of a large survival benefit in meta-analyses.^7^,^8^ Evidence suggests that only a subset of patients will benefit from this therapy. Nearly all patients experience side effects, including fatigue, fever, arthralgia, anorexia, hepatic toxicity, and depression that can be severe. A better understanding of the mechanism of action of IFN-α2b and identification of predictive markers that would permit selection of patients most likely to benefit would therefore be beneficial.

Serological typing for both human leukocyte antigen (HLA) class I and class II antigen expression has previously shown an association between specific HLA antigen expression and clinical response to therapy or OS of patients with metastatic melanoma treated with interleukin (IL)-2 (eg, HLA-DQ1)^9^,^10^ or combinations of IFN-α and IL-2.^11^ One report demonstrated an association of homozygosity of HLA-DR and decreased chance of response to treatment with IL-2.^12^ The purpose of the present study was to evaluate the role that HLA class I or major histocompatibility complex I (determined at low resolution) and HLA-DR/DQ or major histocompatibility complex II (determined at high-resolution) might exert on the outcome of high-risk melanoma patients receiving adjuvant high-dose interferon. The patients included in this analysis were a subgroup of patients enrolled in a randomized phase 3 trial conducted by the Hellenic Cooperative Oncology Group to evaluate intravenous induction therapy with interferon alfa-2b for 4 weeks as compared with the same regimen followed by 11 months of adjuvant interferon alfa-2b therapy.

In addition, we compared HLA frequencies in melanoma patients and healthy controls (healthy unrelated individuals from the Donor Marrow Registry of the National Tissue Typing Center, Athens, Greece). Furthermore, because an increased risk of developing disease recurrence as well as second primary melanomas in melanoma patients presenting with localized disease has been associated with HLA-DQB1*0301 or HLA-DRB1*1101 alleles,^13-16^ we compared the frequency of these genotypes in 2 groups of melanoma patients defined by outcome—namely, those who remained free of recurrence and those who have experienced disease recurrence. HLA alleles related to the development of autoimmune diseases^17^ were studied as a consequence of our observation of correlations between autoimmunity and disease outcome on treatment with IFN.

## MATERIALS AND METHODS

### Patients

Blood samples were collected with ACD anticoagulant from 284 melanoma patients and a panel of 246 randomly selected healthy unrelated Greek individuals, who served as a control population.

Patients participating in this study were enrolled in Trial 13A/98, a prospective, multicenter, randomized phase 3 trial conducted at 13 institutions by the Hellenic Cooperative Oncology Group. In this trial, 364 patients with histologically documented AJCC stage IIB, IIC, or III primary cutaneous melanoma were enrolled between 1998 and 2004. For patients with clinically negative lymph nodes, stage was defined pathologically using sentinel lymph node (SLN) biopsy. Any patient with a positive SLN was required to undergo complete lymphadenectomy. All patients were randomized to receive protocol treatment within 2 months of initial surgery or 1.5 months of therapeutic lymph node dissection. The regimen used was a modification of the E1684 regimen.^6^ Group A patients received IFN-α2b (15 MIU/m^2^/d intravenously 5 days per week for 4 weeks) followed by observation. Group B patients received the same induction dose for 4 weeks followed by subcutaneous therapy (10 MIU/day 3×/wk) for an additional 48 weeks. The primary endpoint for the core protocol was RFS and OS by treatment group.

The HLA substudy reported here was conducted prospectively at 4 institutions participating in the core protocol. This substudy had separate institutional review board approval, and all patients provided written informed consent to this substudy. Blood samples for evaluation of HLA were drawn at the same time as samples for routine initial visit clinical tests. The first 10 mL of blood collected was used for standard biochemistry and blood cell counts, and the second 3 mL was used for HLA testing. Blood samples were obtained before treatment.

The clinical outcome of patients was prospectively followed using standardized testing. Clinical staging consisted of medical history, physical exams, blood cell counts, and blood biochemistry at 3-month intervals, and chest x-ray and liver ultrasound at 6-month intervals.

### Methods

#### DNA extraction

Genomic DNA was extracted and isolated from whole peripheral blood of all patients and controls, using magnetic beads and Biorobot EZ1 (an automated instrument for nucleic acid purification) and a genetic extraction kit, following the manufacturer's protocol (Genovision, Oslo, Norway; Qiagen, Hilden, Germany). DNA was dissolved in doubly distilled H_2_0, DNA concentration was estimated by measurement of OD260, and purity was measured by the ratio of OD260/280. The final preparation was stored at −20°C until further analysis.

#### HLA Typing

Genomic HLA typing was performed using previously published DNA based techniques.^18-20^ Initially HLA-A, -B, -Cw, -DRB1, and -DQB1 low resolution molecular typing was performed in all subjects, with amplification of genomic DNA by polymerase chain reaction (PCR) using locus-specific primers and reverse hybridization with sequence and allele-specific oligonucleotide probes, using a commercially available kit (Lambda Array Beads Multi-Analyte System, LABType RSS0, One Lambda, Inc., Canoga Park, Calif). Subsequently, high-resolution typing at the allele level of HLA-DRB1 and -DQB1 loci was implemented by PCR using sequence-specific oligonucleotides (ELPHA HiRes, Biotest, Dreieich, Germany) and PCR with sequence-specific primers (Olerup SSP, Saltsjoebaden, Sweden), respectively, following the manufacturers' recommendations. The allele assignment was made according to the HLA-visual software program.

### Statistical Analysis

Allele frequencies were defined with 2 different approaches. In the first approach, each individual was used as a unit, and a particular allele was noted as present if detected at least once in an individual. In the second approach, the total number of detections of a particular allele was counted, that is, the allele presence was counted once in heterozygous and twice in homozygous phenotypes. In this case, the allele frequency was calculated over twice the number of individuals tested. Specific allele frequencies were calculated for both the patient population and the healthy control population. Fisher exact test was used for comparing the frequency of specific alleles (1 observation per patient) between the healthy and patient populations as well as the frequency of recurrence between the population in which the specific allele was present versus the population in which it was absent.

In addition, recurrence and specific allele frequencies were compared between patients with or without development of autoimmunity.^21^ Development of autoimmunity was defined as either a positive test for autoantibodies or presentation with a clinical manifestation of autoimmunity in the 12-month period during which blood samples were analyzed for autoantibodies. Two-sided *P* values were used with no adjustment to account for multiple comparisons. The analyses presented here should be considered as exploratory and hypothesis generating rather than conclusive. The relationship between presence of specific alleles, autoimmune response (landmark analysis at 12 months), and patient RFS and OS were further studied using Cox proportional hazard models. The landmark analysis includes patients without event up to 12 months and still on follow-up at 12 months. They are classified by whether they had autoimmunity, based on their autoimmunity status at 12 months, that is, after the last evaluation of autoimmunity was performed.^21^ Survival was evaluated from the date protocol treatment was started to the date of last follow-up or date of death from any cause. RFS was calculated from the initiation of treatment to the date on which relapse was first documented or on which death without documented relapse occurred. The reverse censoring method was used for calculating descriptive statistics; for the follow-up time, Kaplan-Meier estimates of RFS and OS are presented.^22^ For all alleles presented, the ones with frequency <5% in the melanoma population are not included in the analysis.

Alleles defined a priori were: HLA-A*02, HLA-B*35, HLA-Cw*04, HLA-Cw*06, HLA-Cw*07, HLA-DQB1*0301, HLA-DQB1*0501, *0502, *0503, *0504, HLA-DQB1*0601, *0602, *0603, *0604, *0605, *0608, *0609 (HLA DQB1*05 and HLA DQB1*06 correspond to previously serologically typed HLA-DQ1), HLA-DRB1*1101, HLA-DRB1*03, and HLA-DRB1*04. The possibility of chance deviation still exists without correction, and for this reason we consider *P* values between .01 and .05 as probably significant, as suggested by others.^23^ SAS 9.1 (SAS Institute, Cary, NC), was used for statistical analysis, whereas RFS and OS curves were generated with SPSS version 15.

## RESULTS

The frequency patterns of HLA class I and II were first evaluated in the healthy control and melanoma populations. Eleven HLA alleles were found to be significantly different between the melanoma patients and the healthy control population. For the remainder, no significant differences between the distribution of HLA genotype were found in the melanoma population compared with the healthy control population. The alleles that differed among melanoma patients were HLA-A*03, HLA-B*037, *53, *54, *58, *59, *78, HLA-DRB1*1102, HLA-DQB1*0201, and *0302 with corresponding *P* values .042, .033, .050, .046, .001, .021, .004, .021, .006 and .033, respectively. Most of these alleles are represented in <10% in both populations.

Patient demographics and baseline characteristics have been described elsewhere.^6^,^21^ With a median follow-up of 70.67 months (only among patients alive [censored values], range, 7.1-138.7 months), there were 156 recurrences and 104 deaths. The association of HLA genotype with absence of recurrence is presented in Table 1. Correlations between nonrecurrence (alive/well vs recurrent/dead) and the presence of MHC class I allele HLA-Cw*06 and MHC class II allele HLA-DRB1*1501 were noted with the corresponding *P* values .035 and .045, although these are not corrected for multiple testing. When we examined the gene frequency, no further information was acquired on the predictive value of HLA. This was the case also in comparing gene frequency between healthy controls and melanoma patients.

**Table 1:** Association Between HLA Genotype and Recurrence in High-Risk Melanoma Patients Receiving Adjuvant Interferon (Median Follow-up, 71 Months)

HLA-A	No Evidence of Recurrence, n = 128		Recurred, n = 156	
	No.	%	No.	%
Class I				
A*01	25	19.53	39	25.00
A*02	60	46.88	73	46.79
A*03	14	10.94	16	10.26
A*11	13	10.16	20	12.82
A*23	6	4.69	13	8.33
A*24	40	31.25	39	25.0
A*26	18	14.06	14	8.97
A*30	8	6.25	9	5.77
A*32	15	11.72	27	17.31
A*33	11	8.59	11	7.05
A*68	8	6.25	7	4.49
HLA-B				
B*07	11	8.59	14	8.97
B*08	8	6.25	13	8.33
B*13	12	9.38	6	3.85
B*15	4	3.13	12	7.69
B*18	34	26.56	36	23.08
B*35	33	25.78	50	32.05
B*37	7	5.47	8	5.13
B*38	11	8.59	5	3.21
B*39	12	9.38	10	6.41
B*40	8	6.25	11	7.05
B*44	18	14.06	19	12.18
B*51	24	18.75	44	28.21
B*52	7	5.47	9	5.77
B*55	9	7.03	12	7.69
B*57	8	6.25	6	3.85
B73	1	0.78	1	0.64
HLA-C				
Cw*01	8	6.25	10	6.41
Cw*02	11	8.59	23	14.74
Cw*03	19	14.84	20	12.82
Cw*04	36	28.13	49	31.41
Cw*05	9	7.03	11	7.05
Cw*06	32	25	23	14.74
Cw*07	52	40.63	61	39.10
Cw*12	36	28.13	37	23.72
Cw*14	5	3.91	11	7.05
Cw*15	17	13.28	16	10.26
Cw*16	7	5.47	8	5.13
Class II				
HLA-DRB1				
DRB1*0101	11	8.59	16	10.26
DRB1*0301	17	13.28	22	14.10
DRB1*0701	23	17.97	19	12.18
DRB1*1101	19	14.84	27	17.31
DRB1*1104	46	35.94	54	34.62
DRB1*1301	15	11.72	10	6.41
DRB1*1302	8	6.25	12	7.69
DRB1*1401	9	7.03	14	8.97
DRB1*1501	10	7.81	25	16.03
DRB1*1502	6	4.69	8	5.13
DRB1*1601	38	29.69	36	23.08
HLA-DQB1				
DQB1*0201	18	14.06	24	15.38
DQB1*0202	21	16.41	16	10.26
DQB1*0301	68	53.13	82	52.56
DQB1*0302	9	7.03	13	8.33
DQB1*0501	23	17.97	26	16.67
DQB1*0502	43	33.59	45	28.85
DQB1*0503	9	7.03	18	11.54
DQB1*0601	6	4.69	11	7.05
DQB1*0602	9	7.03	13	8.33
DQB1*0603	15	11.72	11	7.05
DQB1*0604	7	5.47	10	6.41

HLA indicates human leukocyte antigen.

The alleles A*25, A*29, A*31, A*66, A*69, A*80, B*14, B*27, B*41, B*45, B*47, B*49, B*50, B*53, B*56, B*58, Cw*08, Cw*17, DRB1*0102, DRB1*0401, DRB1*0402, DRB1*0403, DRB1*0405, DRB1*0408, DRB1*0801, DRB1*0802, DRB1*0803, DRB1*0804, DRB1*1001, DRB1*1103, DRB1*1201, DRB1*1303, DRB1*1321, DRB1*1404, DRB1*1407, DRB1*1503, DRB1*1602, DQB1*0302, DQB1*0303, DQB1*0305, DQB1*0402, DQB1*0504, DQB1*0605, and DQB1*0609 with frequency <5% in the melanoma population are not included in the analysis.

**Figure 1:**
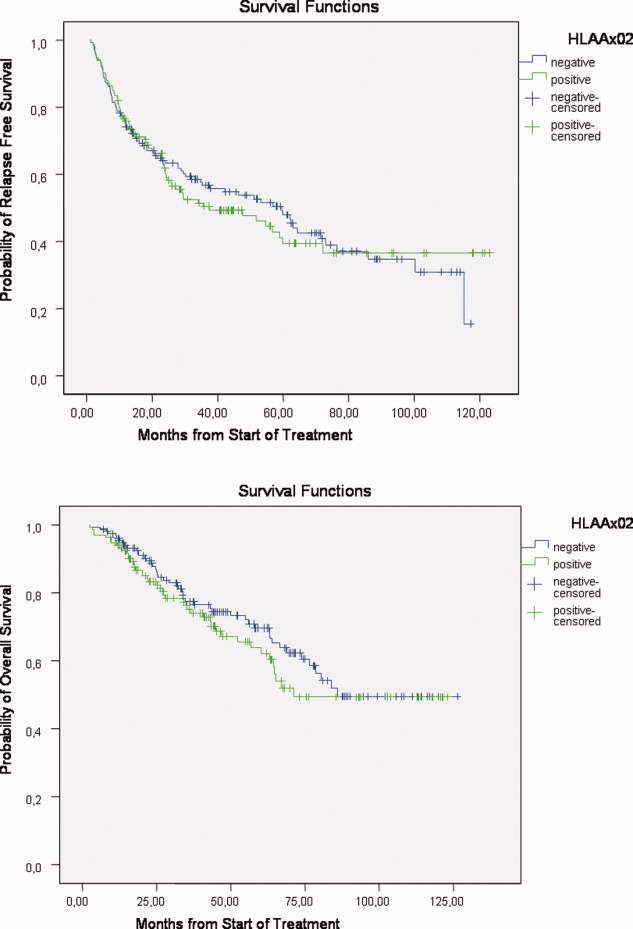
*(Top)* Relapse-free survival plot shows human leukocyte antigen (HLA)-A2 status (positive, n = 133; negative, n = 151). *(Bottom)* Overall survival plot shows HLA-A2 status (positive, n = 133; negative, n = 151).

**Figure 2:**
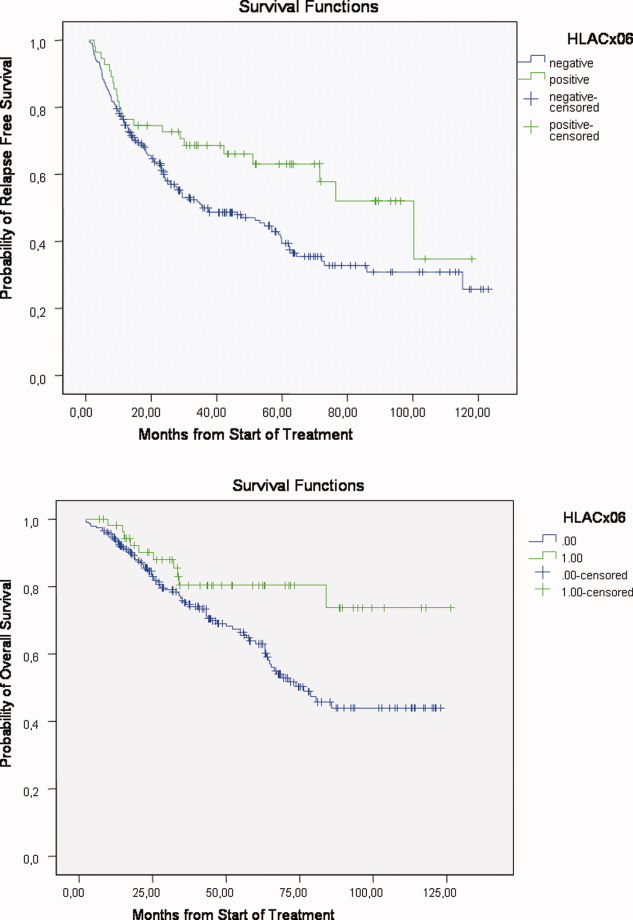
*(Top)* Relapse-free survival plot shows Cw6 status (positive, n = 55; negative, n = 229). *(Bottom)* Overall survival plot shows Cw6 status (positive, n = 55; negative, n = 229). HLA indicates human leukocyte antigen.

Comparison of RFS and OS for previously defined alleles of interest also showed no statistically significant difference among the other alleles investigated (A*02, B*35, Cw*04, Cw*07, DQB1*0301, DRB1*1101, DRB1*03, DRB1*04, DQB1*05 + *06) with the exception of Cw*06. The median RFS of the Cw*06-positive cohort was 100.2 months (range, 2.7-100.2; 95% confidence interval [CI] not evaluable) versus 37.3 months in the Cw*06-negative cohort (range, 1.1-115.1; 95% CI 21.1-53.5; P = .013). The median OS of the Cw*06-positive cohort has not yet been reached (observed minimum and maximum death times 9.8 and 84.2 months; 95% CI not evaluable) versus 78.9 months in the Cw*06-negative cohort (range, 2.3-86.1; 95% CI not evaluable), with a *P* value of .025. Apart from HLA-Cw*06, stage was found to be an independent predictor for RFS (*P* = .029) and OS (*P* = .013) in univariate analysis. The association of HLA-Cw*06 with RFS and OS was explored through a multivariate Cox model including lymph node involvement, ulceration, and stage. In the presence of HLA-Cw*06 in the Cox model, only stage was found to be an independent predictor for RFS (*P* = .010) and OS (*P* = .0011). Controlling for disease stage, the *P* values for the association of Cw*06 with RFS and OS are .018 and .039, respectively. Treatment duration and interaction of treatment duration by HLA-Cw*06 were found nonsignificant, when included in the multivariate Cox models for RFS and OS (*P* = .20 and *P* = .46, respectively for RFS; *P* = .33 and *P* = .86, respectively for OS), along with HLA-Cw*06 and autoimmunity. The Kaplan-Meier curves for RFS and OS in the A*02 and Cw*06 cohorts are presented in Figures 1 and 2. The median OS of the B*35-positive cohort was 69.2 months (range, 8.0-86.1; 95% CI, 49.3-89.1) and has not yet been reached for the B*35-negative cohort (range, 2.3-84.2; 95% CI not evaluable), with *P* = .040 and *P* = .1383 when controlling for disease stage. No differences were observed in RFS (*P* = .143 and *P* = .2141 when controlling for disease stage).

In the cohort of patients included in the prospective autoimmunity study, allele frequency was investigated in 155 of 200 patients (47 in the autoimmunity group and 108 in the nonautoimmunity group). HLA-Cw*06 was expressed in 29.79% (14 of 47) of patients in the autoimmunity group and 15.38% (16 of 108) of patients in the nonautoimmunity group (*P* = .049). Statistical significant differences were seen in HLA-B*35 (21.28% vs 41.35%; *P* = .018) and HLA-DRB1*0701 (23.4% vs 7.69%; *P* = .015, respectively). No differences were seen in the expression of HLA-A*02 (31.91% vs 48.8%; *P* = .077).

In the landmark model for the association of Cw*06 with RFS, Cw*06 and autoimmunity were statistically significant (*P* = .020 and *P* < .0001, respectively). In the landmark model for the association of Cw*06 with OS, only autoimmunity was statistically significant (*P* < .0001). In the landmark models for the association of HLA-B*35, HLA-DRB1*0701, and HLA-A*02 with RFS or OS, only autoimmunity was found statistically significant (*P* < .0001). For all other cases, interaction between alleles and autoimmunity was also tested, but it was found to be insignificant.

## DISCUSSION

This prospective analysis was performed to evaluate whether HLA genotype influences the likelihood of a favorable outcome or response to IFN adjuvant therapy. To answer these questions, it was first necessary to define a baseline population for comparison. No database was available that describes the prevalence of HLA alleles among Greek melanoma patients. Several reports have analyzed the HLA genotypes in North American Caucasian, Italian, Spanish, and Scandinavian populations, yielding conflicting data.^10^,^24-26^ No significant differences were seen in the HLA profiles of the Greek healthy control and melanoma populations studied here.

In the E2690 laboratory corollary of the intergroup adjuvant trial E1690, among patients expressing HLA-A*02, IFN-α2b treatment had a lesser impact on RFS (*P* = .02) compared with the observation arm; among patients not expressing HLA-A*02, the RFS was better for IFN-treated patients.^27^ The differences did not reach significance for RFS among patients in the HLA-A*02-negative subset because of the small size in the subset analyzed from trial E1690 (*P* = .16). The test for interaction between treatment and HLA-A*02 status was marginally significant (*P* = .06). Therefore, this apparent interaction may be a statistical artifact. In our group of patients, the outcome of HLA-A*02-positive patients did not differ from the HLA-A*02-negative cohort in terms both of RFS and OS. Although patients in these 2 studies are of different ethnic backgrounds (North American Caucasian and Southern European Caucasian), and the methods used for HLA typing differed (serological in the E2690 and molecular in the present), the percentages of HLA-A*02 are similar; 42.7% and 46.8%, respectively. Likewise, other class I alleles such as A*11, or class II alleles such as HLA-DQ1, an allele related to the susceptibility to autoimmune diseases,^28^ initially found to be associated with clinical response and survival with IL-2 therapy in the metastatic setting,^9^,^10^ were not found to be related to absence of recurrence in our group of patients. Interpretation of the results in the IL-2–treated patients should take into consideration the caveats associated with retrospective studies involving a relatively limited number of patients not homogenously treated along with uncorrected statistical correlations. Similarly, in our study there are potential weaknesses in identifying differences in outcome based on HLA status, possibly including a subset of patients participating in a randomized trial in which half of the patients received 1 month and half 1 year of adjuvant interferon, as well as the mixed-stage patient population. In addition, the large number of tests performed exploring possible associations between HLA and disease, recurrence, and autoimmune response would lead to some significant results occurring by chance without biological significance. Thus, the analyses presented here should be considered as exploratory and hypothesis generating rather than conclusive. Moreover, in an updated report on 272 patients treated with IL-2–based therapy at the National Institutes of Health, no significant association between HLA type and response was observed.^12^ However, in other immune strategies for melanoma such as polyvalent melanoma, cell vaccine OS after treatment correlated with HLA phenotypes.^29^ However, it is important to mention that both resolution and reliability of molecular HLA typing has substantially improved during the last decade, which has led to partial revisions in HLA associations.

Similarly, no differences were seen when patients were divided according to the presence or absence of HLA-B*35, HLA-Cw*04, HLA-Cw*07, HLA-DRB1*301, HLA-DRB1*04, or HLA-DRB1*1101, alleles associated with thyroiditis (postpartum thyroiditis, Hashimoto disease), rheumatoid arthritis, Sicca syndrome, and diabetes,^17^ or HLA-DQB1*0301, previously correlated with recurrence in stage I and II melanoma patients. We investigated HLA alleles that are correlated with a higher risk of specific autoimmune diseases, as we have previously shown that the appearance of autoantibodies and/or clinical signs of autoimmunity are strongly associated with improved RFS and OS in patients with melanoma who are receiving adjuvant therapy with high-dose IFNa2b.^23^ Failure to demonstrate the association of thyroid autoimmunity with certain HLA types in this study might in part be explained by the finding that their presence increases the probability by no more than 3-fold in most cases.^17^ Moreover, this observation suggests that the IFNa2b-related induction of autoimmunity in melanoma patients differs from spontaneously occurring autoimmune disorders with respect to HLA genetics and presumably also in other aspects of this multifactorial process. This is not surprising, because the current understanding of the development of thyroid autoimmunity suggests a T-cell–mediated disease with mostly unknown multifactorial etiology, such as infection or chronic inflammation with a strong genetic component.^30^ Thus, different routes of autoimmunity may be associated with different sets of genes. Nevertheless, it is most interesting that HLA-Cw*06-positive patients have better RFS and OS (*P* = .013 and *P* = .025, respectively), which remains statistically significant for RFS even in the presence of autoimmunity (*P* = .020). In addition, a statistically higher percentage of Cw*06-positive individuals was present in the cohort of patients who developed autoimmunity. Although the Cw*06 allele has been correlated with psoriasis, the IFNa2b-related autoimmunity was not limited to the skin, affecting other organs as well.

As none of the HLA class II alleles showed significant positive or negative association with the exception of Cw*06, this result is presented here to allow others to consider the evaluation of this issue in trials of IFN-α2b that are being conducted in separate US and European cooperative groups. In particular, as Cw*06-positive patients may have slower-growing melanomas irrespective of IFN therapy, this allele may thus be a prognostic rather than a predictive marker. A validation set of non–IFN-treated melanoma patients should be examined to evaluate this hypothesis. The exploration of this finding in melanoma patients matched for HLA type and adjusted for stage from data sets of randomized controlled trials with an observation arm would answer this question. A final question arises in regard to the correlation of Cw*06 and favorable prognosis of melanoma: the expression of this allele is also correlated with the development of psoriasis, a proinflammatory disorder of the skin in which patients are intolerant of IFNα. It was recently shown that the fraction of patients who benefitted from IFN therapy in the randomized intergroup trial E1694 and were alive at 5 or more years correlates with elevated pretreatment blood serum levels of IL-6, tumor necrosis factor α, IL-1α, and IL-1β.^31^ The correlation of Cw*06 and elevation of proinflammatory cytokines is thus a new topic of interest in view of the findings reported in this study.

## CONFLICT OF INTEREST DISCLOSURES

This study was supported by the Hellenic Cooperative Oncology Group, the National Tissue Typing Center, Athens, Greece, and Award number P50CA121973 from the National Cancer Institute. Helen Gogas, John Kirkwood, and Vernon Sondak have served as consultants to Schering Plough. John Kirkwood and Vernon Sondak have received honoraria from Schering Plough.
